# High Prevalence of Varus Knee Malalignment in Adolescent Football Players—Clinical Lower Leg Axis Measurements of Male Junior Football Players Aged 7 to 18 Years

**DOI:** 10.3390/children11080953

**Published:** 2024-08-07

**Authors:** Clemens Memmel, Dominik Sporrer, Dominik Szymski, Johannes Weber, Alexander Hanke, Markus Denzinger, Maximilian Kerschbaum, Volker Alt, Werner Krutsch, Matthias Koch

**Affiliations:** 1 Department of Pediatric Surgery and Orthopedics, Clinic St. Hedwig, Barmherzige Brueder Regensburg, KUNO Pediatric University Medical Center, 93049 Regensburg, Germany; 2 Department of Trauma Surgery, University Medical Center Regensburg, 93053 Regensburg, Germany; 3 FIFA Medical Center of Excellence, University Medical Center Regensburg, 93053 Regensburg, Germany

**Keywords:** varus knee, bowlegs, junior football, lower leg alignment, training load

## Abstract

Background: Football, as the most popular sport worldwide, has long been under suspicion of causing varus knee alignment as early as adolescence. However, no causal relationship has yet been found. The first step to do so would be to determine the prevalence of lower leg malalignment among male junior football players depending on age, performance level and the number of active seasons played. Methods: Leg axis alignment in frontal plane was determined in male junior football players of different age levels between 7 and 18 years by measuring the intercondylar/intermalleolar distance (ICD/IMD) in an upright position. In addition to anthropometric data, multiple sport-specific data such as the start of their football career or training time per week were collected by means of questionnaires (clinical trial registration number: DRKS00020446). Results: 207 male junior football players were included in this survey. The mean age was 12.8 years. Within the group of 15 to 18 year olds, the prevalence of varus knee malalignment was highest at just under a third (32.1%). In the subpopulation that played actively for more than ten seasons, 28.9% showed varus leg axis. Regarding performance level, the highest IMD/ICD values and highest prevalence of varus alignment were found among football players who play on a semi-professional level (16.7%), compared to amateur (11.4%) and high-performance levels (2.8%). Conclusions: Further research is necessary to investigate if this high prevalence of varus knee in children of a higher age and higher playing levels is due to the selection bias of football players with varus knee or a real development of varus knee in individuals.

## 1. Introduction

In large parts of the world, football is by far the most popular sport in the world and is becoming increasingly popular with children and young people due to media interest. It inspires millions of young and adult athletes of every gender and age [1,2]. The majority of football players begin their football career a long time before the onset of puberty. Thus, practicing football is a constant influence on their overall development during the growing age [1,2]. Besides ball skills and tactical thinking, a football player gets trained in physical skills such as endurance, agility, and speed to meet the demands of the sport [3,4]. These requirements cause increased stress, especially on the lower extremity. Prior studies indicated a higher prevalence of leg malalignments in football players compared to the normal population or other team sports like handball, basketball, or volleyball [5,6,7]. Nevertheless, even if no causal relationship could be established between frequent football play in childhood and adolescence and the prevalence of varus leg malalignment yet, a positive correlation was shown [5,7]. Multiple possible reasons for the development of bowlegs in junior football, such as recurrent microtrauma to the growth plates [8], a muscular disbalance of the lower extremity [9], or specific movements such as shooting for a goal [10], have already been discussed. However, while the definitive causes of the development of leg malalignment are still unclear, there is significant knowledge concerning the development of osteoarthritis in the case of leg malalignment [11,12].

Regarding the current literature concerning osteoarthritis in athletes, in comparison to other sportspeople, particularly football players are at risk of developing knee osteoarthritis [13,14,15]. Up to 80% of former professional football players are affected by knee osteoarthritis [16,17]. A previous major injury of the lower extremity is considered to be the main attributable risk factor in male elite football players [18]. However, studies have shown that even after the adjustment of recognizable risk factors such as major knee injury or training load, knee osteoarthritis appears to be an occupational hazard of professional football [19]. In this context, other investigations showed not only a high percentage of osteoarthrosis among former professional football players but also a percentage of 55–81% of varus knee in the evaluated cohort [16,20].

Considering these findings and respecting the fact that football is a major popular sport with millions of junior and adult athletes, this emphasizes the importance of scientific approaches to analyze a possible connection between varus leg axis development and the practice of football in childhood and adolescence, while the growth plates around the knee joint are extremely vulnerable for the impact of sport-related stress [21]. As the first step for the development of preventive strategies, the definition of the prevalence and severity of lower leg malalignment is necessary [22]. Therefore, the aim of the present study was the determination of the prevalence of lower leg alignment among various sub-populations of junior football players depending on age, performance level and the number of active seasons played.

## 2. Materials and Methods

### 2.1. Design

The local ethics committee (code: 19-1571-101) approved the design and the methods of this project prior to its start. Additionally, the study was registered in the German Clinical Trials Register DRKS (clinical trial registration number: DRKS00020446). Written consent concerning participation, measurement, data acquisition, and the publication of the data was collected for each participant. Before the measurement of each subject, written consent of the subject, and in the case of underage participants, written consent from their legal guardians, was obtained. The consent form included not only their participation in the study but also their agreement on their measurements, the obtaining of additional sport-specific data and finally on the publication of the data. A two-part cross-sectional study design was chosen to analyze the anthropometric and sport-specific data by a questionnaire and the knee alignment by established intercondylar/intermalleolar distance (ICD/IMD) measurements. The consent forms, study guidelines and questionnaire were handed out to the subjects who met the inclusion criteria and were then collected by the study personnel. The survey included questions regarding the subject’s anthropometric characteristics as well as their position in the field, dominant leg, training and match history, level of performance and injury history. The body mass index (BMI) was measured, adjusted for sex and age after the collection of data.

### 2.2. Study Population

The study population consists of a series of *n* = 207 male junior football players aged between 7 and 18 years. Inclusion and exclusion criteria are defined as presented in Table 1. Quota sampling was used to select the study subjects. The study personnel was instructed to select the participants within the age range of 7 to 18 years as well as being equally distributed throughout every performance level (amateur to professional level). Based on age (xage), performance level (xPL), and football exposure (xFE), the participants were respectively divided into three groups (see Table 2). The age groups were categorized according to an estimate of their pubertal growth phase [23] and thus into the age groups 7–11 years (group^age^ I: prepubescent), 12 to 14 years (group^age^ II: pubescent), and 15 to 18 years (group^age^ III: adolescent).

### 2.3. Analysis of the Leg Axis

Lower leg alignment in the frontal plane was assessed via ICD and IMD in a standing position. The subjects were asked to stand upright and shoulder-width apart and then were instructed to move their legs toward each other with small steps until either the malleoli or the medial femoral condyles touched. This procedure was rehearsed three times before each measurement to ensure a standardized and accurate procedure. The next step was to set an orthograde positioning of the patella, in particular to eliminate tibial internal rotation as a confounder of the measurement method [24]. The ICD and IMD [mm] were measured with a digital calliper. The technique has been previously described and established in many studies [5,7,25,26]. Malalignment was defined according to Shohat et al. (2018). Pathological valgus leg axis was defined as IMD > 4 cm. An ICD > 3 cm was noted as a pathological varus malalignment [27].

### 2.4. Data Analysis

Before conducting the measurements, a sample size calculation was performed. With the presented sample size, small to medium effect sizes with a power of 80% at an alpha level of 5% can be detected. Statistical analysis was performed using SPSS^®^ (Version 25, IBM, Armonk, NY, USA). Data are presented as mean ± SD or absolute and relative frequencies. Prior to statistical analysis, variables were checked for normal distribution via the Kolmogorov–Smirnov test, which showed a non-normal distribution for ICD/IMD values. Continuous data between two or more groups were therefore compared with non-parametric tests, in this case, the Kruskal–Wallis test (H-test). In the case of proof of a normal distribution (e.g., the subjects’ age), Student’s *t*-test and an analysis of variance (ANOVA) were performed as a parametric test. A probability (*p*) value of ≤0.05 was considered to be significant for each test. Correlation coefficients were measured using Pearson’s *r.* Graphical illustrations were generated with GraphPad Prism^®^ (Version 5.01, GraphPad Software, La Jolla, CA, USA) and Microsoft PowerPoint 2013^®^ (Microsoft Corporation, Redmond, WA, USA).

## 3. Results

*N* = 207 male junior football players were included in the present study. The overall mean age was 12.8 ± 2.8 years and mean BMI was 19.2 ± 3.1 kg/m^2^. The overall study population’s mean ICD/IMD was –4 ± 33 mm. The Pearson’s correlation coefficient between ICD/IMD and age was *r* = 0.43. In terms of age, weight, and height, the three age groups I–III differed significantly. Regarding BMI, only groupage III differed significantly from groups^age^ I and II (*p* < 0.001, see Table 3). The mean training time varied significantly among groups^age^ I and II (*p* < 0.001) and II and III (*p* = 0.01) but not within groups I and III (*p* = 0.35).

**Figure 1:**
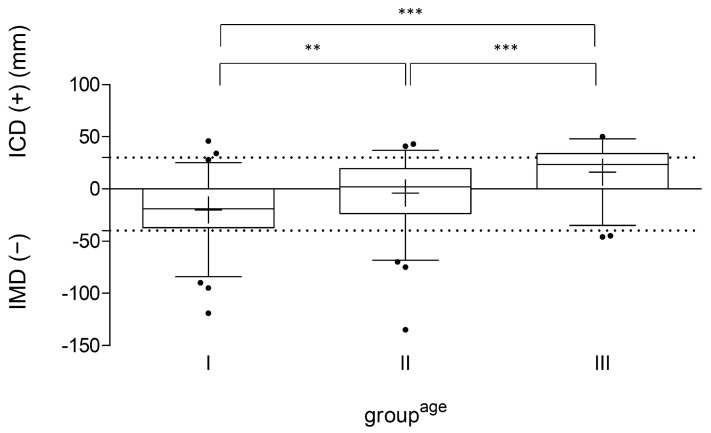
ICD/IMD measurement of all participants (*n* = 207) in their age group (I: 7–11; II: 12–14; III: 15–18 years), indicated in millimeters (mm). The line in the box shows the median, the plus sign indicates the mean value, the whiskers mark the 5th and 95th percentiles, and the dots are individual values that deviate from the 5th and 95th percentiles. The dashed lines mark the border to pathological varus/valgus values. IMD: intermalleolar distance; ICD: intercondylar distance; ** *p* < 0.01; and *** *p* < 0.001.

The analysis of the leg axis of all participating football players showed valgus alignment (IMD ≥ 4 cm) in *n* = 25 players (12.1%) and varus alignment (ICD > 3 cm) in *n* = 26 athletes (12.6%). The mean ICD/IMD values according to groups^age^ I–III are presented in Figure 1. The mean ICD/IMD value of group A was −20 ± 31 mm, of group B −4 ± 31 mm, and of group C 16 ± 24 mm. Significant differences within the groups (A vs. B: *p* = 0.002; A vs. C: *p* < 0.001; B vs. C: *p* < 0.001) were detected. Table 4 shows the percentage of lower leg alignment within the age-related subgroups.

Thigh circumference did not vary significantly between the dominant and nondominant leg within the study population (*p* = 0.94) and it did also not differ among those with pathological varus leg alignment (*p* = 0.94; see Table 5).

**Figure 2:**
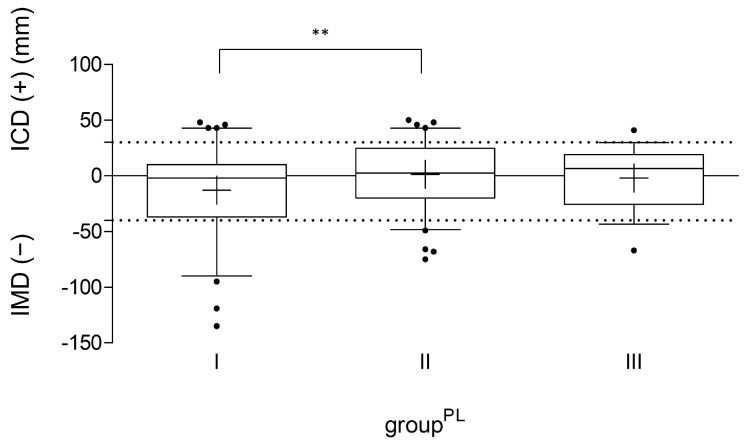
ICD/IMD measurement of all participants with information on training time per week (*n* = 199), divided according to their performance level (group^PL^ I–III), indicated in millimeters (mm). The line in the box shows the median, the plus sign indicates the mean value, the whiskers mark the 5th and 95th percentiles, and the dots are individual values that deviate from the 5th and 95th percentiles. The dashed lines mark the border to pathological varus/valgus values. IMD: intermalleolar distance; ICD: intercondylar distance; ** *p* < 0.01.

*N* = 199 (96.1%) football players provided data according to their performance level. Of those, *n* = 79 athletes (39.7%) trained for up to 180 min per week (basic training exposure, group^PL^ I), *n* = 84 players (42.2%) trained for between three and four 90 min sessions (extended training exposure, group^PL^ II), and *n* = 36 participants (18.1%) trained for more than 360 min per week (high training exposure, group^PL^ III). The ICD/IMD analysis according to the performance level (Figure 2 and Table 6) showed a significantly higher mean ICD/IMD value (*p* = 0.007) and in total a higher prevalence of varus alignment in group^PL^ II in comparison to group^PL^ I. The players in group^PL^ III showed a lower prevalence of both varus and valgus alignment with a comparable mean age. The ICD/IMD values between groups^PL^ II and III as well as groups^PL^ I and III did not differ significantly (*p* = 0.54).

**Figure 3:**
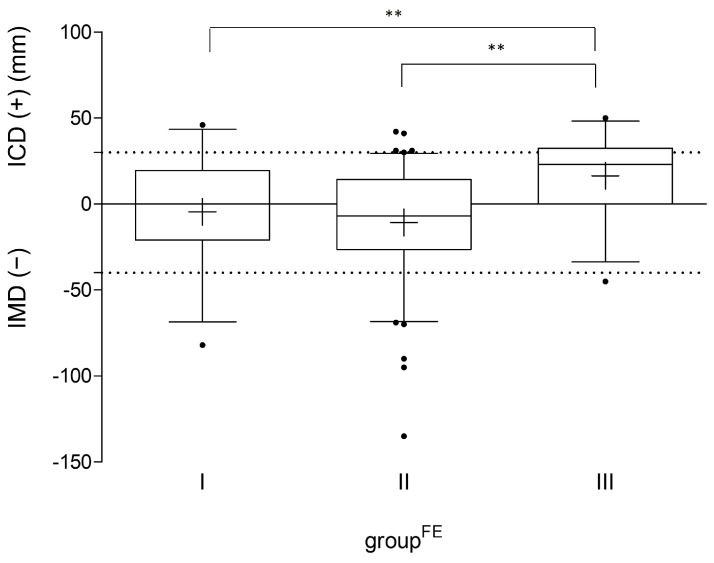
The ICD/IMD measurement of all participants with information on the number of active seasons played (*n* = 185), divided into group^FE^ I (≤5 seasons), group^FE^ II (6 to 10 seasons) and group^FE^ III (>10 seasons), indicated in millimeters (mm). The line in the box shows the median, the plus sign indicates the mean value, the whiskers mark the 5th and 95th percentiles, and the dots are individual values that deviate from the 5th and 95th percentiles. The dashed lines mark the border to pathological varus/valgus values. IMD: intermalleolar distance; ICD: intercondylar distance; ** *p* < 0.01.

In total, *N* = 185 participants (89.4%) provided information about their overall football exposure (first time playing football on a regularly basis and number of active seasons played since then). Regarding the different periods of football exposure (group^FE^ I–III), the players of group^FE^ III had significantly higher ICD/IMD values as well as a higher prevalence of varus knee alignment compared to athletes with less active seasons played (*p* < 0.01, see Figure 3 and Table 7). No significant differences were seen between groups^FE^ I and II.

## 4. Discussion

The present study illustrates quite clearly the exposure time-dependent effect of football on the leg alignment within a junior male football player cohort. In the age group of 15 to 18 year olds, nearly every third football player showed varus knee alignment. Football exposure of 10 years and more were associated with significantly higher mean ICD/IMD values (*p* < 0.01) and the prevalence of varus knee alignment in comparison to the groups with less football exposure (see Table 7 and Figure 3). These findings are in accordance with the results of Isin et al. as well as Cock et al. describing a cumulative effect on the leg axis alignment in adolescent athletes [28]. Isin et al. (2020) showed a strong positive correlation between training years and the IMD of adolescent football players [5]. For weight-bearing sports in general and football in particular, Cock et al. (2018) described a similar positive correlation between the ICD/IMD values and the ‘cumulative sports factor’, a coefficient that includes both the number of years of exercise and the average number of hours a week [28].

Additionally, the evaluation of a cohort of male junior football players facilitates an analysis of a more homogenous group of athletes regarding age, performance level or training load in comparison to an analysis of adolescent football players. Thus, it can be assumed that at least the cumulative effect on leg axis development during growth is not football-specific as a similar effect was also shown for jogging and volleyball in the study by Cock et al. [28]. The cumulative effect is also reflected in the development of ICD/IMD values with increasing age. This progression is described in further studies using the measurement of ICD and IMD to reflect the alignment of the lower extremities [8,9]. Based on this, in the present study, the groups were defined according to the athlete’s growth phase as described before, and the comparison of the ICD/IMD values showed a significant increase with developing growth (see Figure 1). This finding, the development of varus knee alignment influenced by the number of actively played seasons and age, might be explained by the Hueter–Volkmann law, which describes the effect of pressure on epiphyseal growth [29,30,31]. According to Hueter and Volkmann, increased pressure, applied parallel to the axis of an epiphysis, inhibits epiphyseal growth and respectively promotes it in cases of decreased pressure. Consequently, changes in compressive forces cause the asymmetrical growth of a joint [32]. Furthermore, the ‘chondral modelling theory’ by Frost suggests that physiological loading stimulates growth, whereas loads outside this range, either higher or lower, will inhibit it [33,34]. This model, in turn, implies not a linear but a parabolic dose–response principle depending on the ratio and the amount of pathological and physiological stress, respectively, of the open growth plates. This relationship can also be seen in the results of the present study. The evaluation of the performance level showed significantly higher ICD/IMD values and a higher prevalence of pathological varus knee alignment upon comparing performance levels I and II, even if the mean age of both groups differed by only two years. Additionally, a stepwise increase in ICD/IMD values was observed regarding the training exposure (basic–high–extended training exposure). This could be explained by the fact that it is not the training load itself that causes varus malalignment but the training methodology, which has a significant influence on the type and amount of load. Thus, it has to be assumed that football clubs which perform on a high-performance level increasingly focus on balanced training with a higher focus on the prevention of muscular imbalances or training intensity. This hypothesis is supported by the finding of the present study. The percentage of both valgus and varus malalignments was the lowest at the highest performance level. Conversely, this means that 94.4% of the football players of the highest performance level show physiological values.

Concerning the prevalence of varus knee alignment, Asadi et al. (2015) described a higher prevalence of varus knee alignment compared to other weight-bearing sports like basketball, volleyball, or handball [10]. Analogously, Thaller et al. (2018) reviewed that the ICD/IMD values of young football players are higher compared to other sports, i.e., they tend to have varus leg axis more often than in other sports [7]. This, in turn, suggests that it would have to be football-specific movement patterns, e.g., passing or shooting for a goal, that would lead to additional varicosity beyond the basic stress of weight-bearing sports. In particular, the adduction moment could play an essential role in this because the kicking movement represents not only a hip flexion and extension in the knee joint but a much more diagonal movement of the leg towards the midline [35,36]. Witvrouw et al. (2009) discussed the football-specific disequilibrium of the leg muscles to be the main reason [9]. To dig further into these two hypotheses, the thigh circumference among the football players for the dominant and non-dominant leg was measured for this survey to detect possible differences among players with varus alignment and those with physiological knee alignment, but no significant differences could be found. A recent study also showed that adolescent football players have higher values of the hip–knee–ankle angle (more towards varus) than football referees of the same age who had significantly less overall football exposure time during their childhood [37]. This could be another indication of a football-specific impact on the lower leg alignment. In the overall picture, however, it remains still unknown what exactly is the football-specific factor that causes an increased prevalence of varus knee alignment.

Despite all of the strengths and unique data collection of this study, it has some limitations which should be considered in the interpretation of its results. First of all, quota sampling has its disadvantages as it might under- or overrepresent certain subgroups. Ultimately, this type of recruitment is subject to a certain degree of arbitrariness. Furthermore, the categorization into age groups according to the estimated growth phase has its own limitation. Of course, there are individual differences when it comes to the onset of puberty. Secondly, since radiological diagnostics with ionizing radiation (e.g., full leg x-ray) cannot be justified in healthy children, research in this area is fundamentally tied to clinical measurement methods and observational studies. In the case of the ICD/IMD measurement, this causes a lack of differentiation of the leg axis between the dominant and non-dominant leg, which is certainly advantageous for further discussion when it comes to the accessibility of muscular imbalances in the kicking/standing leg as a possible cause of varus leg axis development, although Colyn et al. (2016) could not detect significant differences between the hip–knee–ankle angles of the dominant versus the non-dominant leg [38]. Another weakness of this study is that, due to the study design constituting a cross-sectional analysis, conclusions about causality and effect can only be made to a limited extent and only with regards to correlations. To avoid this limitation, an age-matched control group would be necessary, which could not be realized in this study project. Despite all limitations, the data retrieved from this study provide a reliant basis for future prospective studies.

## 5. Conclusions

The leg axis develops towards varus with age among football players. Furthermore, the highest prevalence of varus leg axis can be found in junior football players aged 15 to 18 and in the population that played more than ten seasons during childhood. Regarding the performance level, the highest IMD/ICD values and prevalence of varus deviations are found among semi-professional football players. Although various studies have shown a positive correlation between football exposure and varus knee alignment, the causality is yet to be proven. This study, in contrast to some others, does not show a linear correlation between training time and the prevalence of varus leg axis but more a dependence on performance level, the number of seasons played, and age. Longitudinal studies might help provide further insights with extended study populations, e.g., with an annual leg axis measurement and retrospective query of football exposure and growth per year. These studies might allow more differentiated conclusions about the effect of football on the development of the leg axis in the frontal plane. Based on these findings, football-specific risk factors can be identified that could be specifically addressed through primary prevention measures in training methodology.

## Figures and Tables

**Table 1:** Inclusion and exclusion criteria of the study population.

Inclusion Criteria	Exclusion Criteria
active member of a football team registered in the Bavarian Football Association aged 7 to 18 years football as the main practiced sport (largest percentage of training and match time per week in comparison to other individual sport activities) male sex complete data set	current injury of the lower extremity chronic orthopedic disorder affecting the leg axis major injury prior to measurement which could affect leg axis (e.g., ACL injury, dislocated fractures of lower extremities which required osteosynthesis i. a.)

**Table 2:** Division of study population according to age (group^age^; * classification according to Prader et al. (1989) [23]), performance level (group^PL^) and football exposure (group^FE^).

Criterion:	Age/Growth Phase		
group^age^	age [years]	phase *	characteristics
I	7–11	pre-pubescent	before the main growth phase
II	12–14	pubescent	onset of puberty, main growth phase of the lower limb in male individuals
III	15–18	adolescent	beginning of growth plates closing, main longitudinal growth is completed
criterion:	performance level		
group^PL^	description		training time per week [min]
I	basic training exposure		≤180
II	extended training exposure		180 < x < 360
III	high training exposure		>360
criterion:	football exposure		
group^FE^	number of seasons [*n*]		
I	≤5 seasons		
II	6 to 10 seasons		
III	>10 seasons		

**Table 3:** General data of the study population (*n* = 207), divided into age groups. kg: kilograms; cm: centimeters; SD: standard deviation. ** *p* < 0.01; *** *p* < 0.001.

Age Group [Years]		*n*	Mean Age/SD [Years]	Median Age [Years]	Weight [kg] (Mean/SD/Range)	Height [cm] (Mean/SD/Range)	BMI [kg/m^2^] (Mean/SD/Range)	Training Time per Week [min] (Mean/SD)	Football Exposure [Active Seasons] (Mean/SD)
I	7 to 11	74	9.9/0.9	10	37.5/8.5/ 21.9–65.3	143/8/ 124–161	18.0/2.9/ 13.7–26.9	229/86	5.7/1.7
II	12 to 14	77	12.8/0.8	13	50.2/10.2/ 32.0–76.0	163/10/ 142–188	18.8/2.7/ 12.1–27.2	287/71	7.6/1.7
III	15 to 18	56	16.6/1.3	17	68.0/10.4/ 46.1–97.4	178/7/ 163–197	21.3/2.6/ 16.1–27.0	243/68	10.9/3.0
*p* values			I/II/III ***		I/II/III ***	I/II/III ***	I vs. II: *p* = 0.09 I vs. III *** II vs. III ***	I vs. II *** I vs. III: *p* = 0.35 II vs. III **	I/II/III ***

**Table 4:** Lower leg alignment according to ICD/IMD measurement within the different groups^age^, indicated as total number (*n*) and percentage.

Age Group [Years]		*n*	Valgus (IMD < 4 cm)	Normal	Varus (ICD > 3 cm)
Overall		207	25 (12.1%)	156 (75.3%)	26 (12.6%)
I	7 to 11	74	15 (20.3%)	57 (77.0%)	2 (2.7%)
II	12 to 14	77	8 (10.4%)	63 (81.8%)	6 (7.8%)
III	15 to 18	56	2 (3.6%)	36 (64.3%)	18 (32.1%)

**Table 5:** Comparison of thigh circumference among participants concerning dominant versus non-dominant leg, indicated in centimeters. *p*-values generated from a two-tailed *t*-test. cm: centimetres; SD: standard deviation; IMD: intermalleolar distance; ICD: intercondylar distance. In total, eight subjects did either not indicate which leg was the dominant one or indicated that they were two-footed, which led to exclusion for this analysis.

Thigh Circumference	*n*	Dominant Leg [cm] Mean/SD/Min–Max	Non-Dominant Leg [cm] Mean/SD/Min–Max	*p*-Value
Overall	199	44.3/6.1/30.5–60.0	44.2/6.2/30.5–60.0	0.94
valgus knee alignment (IMD > 4 cm)	25	47.2/5.7/34.5–59.0	46.8/5.8/34.0–60.0	0.81
varus knee alignment (ICD > 3 cm)	24	47.9/5.6/34.5–57.0	48.0/5.5/34.5–56.0	0.94

**Table 6:** Study population with information on training exposure per week during the current season (*n* = 199) and the corresponding ICD/IMD values, number and percentage of valgus/varus and normal knee alignment. IMD: intermalleolar distance; ICD: intercondylar distance; mm: millimeters; SD: standard deviation.

Group^pl^	I (≤180 Min/Week)	II (180 < x < 360 Min/Week)	III (>360 Min/Week)
*n*	79	84	36
Age [y] (mean/SD)	11.8/3.3	13.8/2.3	12.5/1.7
Training exposure [min] (mean/SD)	180/0	268/19	388/64
ICD/IMD [mm] (mean/SD)	−13/37	1/28	−2/25
Valgus/ normal/ varus (*n* (%))	16 (20.3)/54 (68.4)/9 (11.4)	7 (8.3)/63 (75.0)/14 (16.7)	1 (2.8)/34 (94.4)/1 (2.8)

**Table 7:** Study population with information on age of beginning to play football on a regularly basis and the number of active seasons played since then (*n* = 185) and the corresponding ICD/IMD values, number and percentage of valgus/varus, and normal knee alignment. IMD: intermalleolar distance; ICD: intercondylar distance; mm: millimeters; SD: standard deviation.

Group^FE^	I ≤5 Years	II 6–10 Years	III >10 Years
*n*	37	110	38
Age [y] (mean/SD)	10.6/2.6	12.2/1.7	16.9/1.2
Football exposure [y] (mean/SD)	3.8/1.4	7.7/1.3	12.3/1.0
ICD/IMD [mm] (mean/SD)	–5/31	–11/31	16/22
Valgus/normal/varus (*n* (%))	5 (13.5)/25 (67.6)/7 (18.9)	15 (13.6)/91 (82.7)/4 (3.6)	1 (2.6)/26 (68.4)/11 (28.9)

## Data Availability

The data that support the findings of this study are available upon request from the corresponding author (C.M.). The data are not publicly available due to restrictions, e.g., their containing information that could compromise the privacy of the research participants.
